# Post-surgical abdominal wall endometriosis: a retrospective cohort study on clinical features, ultrasound accuracy and recurrence

**DOI:** 10.3389/fmed.2026.1969114

**Published:** 2026-10-29

**Authors:** Shutong Lin, Lu Yu, Zhenglin Mou, Jiayin Gu, Hong Luo

**Affiliations:** 1 Department of Ultrasound, West China Second University Hospital of Sichuan University, Chengdu, Sichuan, China; 2 Key Laboratory of Birth Defects and Related Diseases of Women and Children (Sichuan University), Ministry of Education, Chengdu, Sichuan, China

**Keywords:** abdominal wall endometriosis, cesarean section, extra-pelvic endometriosis, recurrence, ultrasound

## Abstract

**Background:**

Abdominal wall endometriosis (AWE) is a rare but clinically significant condition; yet studies about its preoperative assessment and surgical outcomes remain limited. Our study aimed to characterize its clinical presentation, evaluate the utility of preoperative ultrasound, and investigate postoperative recurrence.

**Methods:**

We retrospectively analyzed 291 AWE patients who underwent surgical resection. Clinical features, ultrasound-surgical concordance, and factors associated with recurrence were assessed. Lesions were classified into Type I (subcutaneous), Type II (fascial/muscular), and Type III (peritoneal) by infiltration depth, with subgroup comparisons across three types.

**Results:**

Palpable mass (81.8%) and cyclic pain (46.7%) were the predominant symptoms. 99.7% of patients had prior cesarean section (CS), but no significant differences were found between multiple versus single CS groups in infiltration depth (*P* = 0.138), maximum diameter (*P* = 0.101), or lesion multiplicity (*P* = 0.225). Deeper infiltration was associated with older age at diagnosis (*P* = 0.030), longer CS-to-diagnosis interval (*P* = 0.023), larger lesions (*P* < 0.001), more frequent abdominal wall pain (*P* = 0.028), fewer palpable masses (*P* = 0.037), and higher rates of postoperative complications (*P* = 0.002). Preoperative ultrasound agreed well with intraoperative measurements of maximum lesion diameter (ICC=0.872), but its ability to detect myofascial invasion was limited (positive predictive value 88.8%; negative predictive value 44.7%; false-negative rate 22.9%). The overall postoperative recurrence rate was 1.8%. Exploratory Kaplan–Meier analysis suggested a higher recurrence rate in patients with BMI ≥25 kg/m^2^. However, this result should be interpreted with caution given the limited number of recurrence events.

**Conclusions:**

AWE occurred mostly in women with a history of CS, yet the number of prior CS did not influence lesion characteristics. Deeper infiltration is associated with greater surgical complexity and higher postoperative complication rates. While preoperative ultrasound reliably measures lesion size, its assessment of myofascial invasion remains limited. The observed recurrence rate was low after surgical excision in our cohort.

## 1 Introduction

Abdominal wall endometriosis (AWE) is defined as the ectopic presence of functional endometrial tissue within the layers of the abdominal wall, including scar endometriosis secondary to surgery and spontaneous AWE (1). The former type is more common and often accompanies cesarean section(CS), whereas the latter type develops without any previous surgery and tends to appear most often around the umbilicus or groin (1). The prevalence of AWE in reproductive-aged women is estimated at 0.03%–3.5% (2); but with cesarean delivery rates rising globally, the incidence of AWE has grown substantially in recent years (3), highlighting the need to enhance clinical awareness and standardized management.

**Table 1:** Clinical characteristics of patients With AWE, stratified by depth of infiltration.

Characteristic	Overall (*n* = 291)	Type I (*n* = 64)	Type II (*n* = 183)	Type III (*n* = 44)	*P*-value
Baseline characteristics					
Age at diagnosis, years, median [IQR]	34.00 [31.00, 36.00]	33.00 [30.00, 36.00]	33.00 [31.00, 36.00]	35.00 [33.00, 38.00]	**0**.**030**
BMI, kg/m^2^, median [IQR]	21.23 [20.24, 23.57]	21.20 [20.42, 23.41]	21.36 [20.24, 23.58]	21.20 [20.13, 23.48]	0.912
Concomitant ovarian endometrioma, *n* (%)	17 (5.8%)	5 (7.8%)	11 (6.0%)	1 (2.3%)	0.473
Concomitant deep endometriosis, *n* (%)	2 (0.7%)	0	1 (0.5%)	1 (2.3%)	0.325
Cesarean section history					
Number of prior CS, *n* (%)					0.146
1 CS	215 (73.9%)	51 (79.7%)	137 (74.9%)	27 (61.4%)	
≥2 CS	75 (25.8%)	13 (20.3%)	46 (25.1%)	16 (36.4%)	
0 CS	1 (0.3%)	0	0	1 (2.3%)	
Latency period, months, median [IQR]	34 [18, 48]	27 [14.25, 42.5]	34 [18.5, 48]	36 [18.75, 64.5]	0.215
Time from symptom onset to diagnosis, months, median [IQR]	12 [6, 30]	9 [3, 36]	12 [6, 24]	22 [6, 48]	0.115
Time from CS to diagnosis, months, median [IQR]	48 [36, 72]	48 [24, 60]	48 [36, 72]	60 [48, 87]	**0**.**023**
AWE lesion characteristics					
Symptom: palpable mass, *n* (%)	238 (81.8%)	55 (85.9%)	153 (83.6%)	30 (68.2%)	**0**.**037**
Symptom: abdominal wall pain, *n* (%)	62 (21.3%)	11 (17.2%)	35 (19.1%)	16 (36.4%)	**0**.**028**
Symptom: menstrual-related pattern, *n* (%)	136 (46.7%)	31 (48.4%)	81 (44.3%)	24 (54.5%)	0.449
Lesion number, *n* (%)					0.395
Solitary	256 (88.0%)	57 (89.1%)	163 (89.1%)	36 (81.8%)	
Multiple	35 (12.0%)	7 (10.9%)	20 (10.9%)	8 (18.2%)	
Maximum diameter, *n* (%)					**< 0.001**
＜3cm	155 (53.3%)	47 (73.4%)	101 (55.2%)	7 (15.9%)	
≥3cm	136 (46.7%)	17 (26.6%)	82 (44.8%)	37 (84.1%)	
Surgical outcomes					
Mesh placement, *n* (%)	7 (2.4%)	0	4 (2.2%)	3 (6.8%)	0.068
Postoperative complications^a^, *n* (%)	11 (3.8%)	0	5 (2.7%)	6 (13.6%)	**0**.**002**

a Postoperative complications included fever, poor wound healing, and incisional hernia.

IQR, interquartile range; CS, cesarean section; AWE, abdominal wall endometriosis.

**Table 2:** Association between number of cesarean sections and AWE lesion characteristics^a^.

Characteristic	1CS (*n* = 215)	≥2CS (*n* = 75)	*P*-value
Depth of infiltration, *n* (%)			0.138
Type I (subcutaneous)	51 (23.7%)	13 (17.3%)	
Type II (fascial/muscular)	137 (63.7%)	46 (61.3%)	
Type III (peritoneal)	27 (12.6%)	16 (21.3%)	
Lesion number, *n* (%)			0.225
Solitary	192 (89.3%)	63 (84.0%)	
Multiple	23 (10.7%)	12 (16.0%)	
Maximum diameter, cm, median [IQR]	2.50 [2.00, 3.00]	3.00 [2.00, 3.00]	0.101

a One patient with 0 CS was excluded (*n* = 290).

The prevailing hypothesis for AWE pathogenesis is iatrogenic implantation of endometrial tissue into the surgical wound during obstetric/gynecological procedures (3). Similar to eutopic endometrium, the ectopic tissue responds to cyclic hormonal changes, leading to periodic bleeding, fibrosis, and the formation of ectopic nodules. Consequently, the typical clinical presentation of AWE includes abdominal pain and/or a palpable mass that fluctuates with the menstrual cycle (4). In more severe cases, the pain may become persistent and significantly impair life quality. However, atypical presentations are common and misdiagnosis remains frequent. Lesions often confused with hematomas, granulomas, desmoid tumors, or malignancies (5), especially in non-specialist settings (6).

Previous studies have described its clinical features, surgical outcomes, as well as the role of imaging in preoperative assessment (7–12). Recently, growing attention has been directed toward the impact of lesion infiltration depth on surgical difficulty and prognosis, leading to the proposal of a clinical classification system based on the level of abdominal wall involvement (10). However, differences in clinical features and surgical outcomes across infiltration-depth subtypes have not been assessed in a large cohort. The value of preoperative ultrasound has also been rarely discussed.

Against this background, we conducted a retrospective analysis of 291 AWE patients. Specifically, we aimed to: (1) compare clinical features and surgical outcomes across three infiltration-depth subtypes; (2) evaluate the accuracy and limitation of preoperative ultrasound; and (3) investigate recurrence after lesion excision.

## 2 Materials and methods

### 2.1 Study population

The study was approved by the Institutional Review Board of West China Second University Hospital (No. [2026] 344). Informed consent was waived given the retrospective nature of the study. We reviewed the medical records of patients who underwent surgical resection and pathologically diagnosed as AWE between January 2023 and January 2026. Patients who did not undergo surgery, lacked pathological confirmation, or had incomplete clinical data were excluded. Figure 1 illustrated the selection process of 291 patients.

**Figure 1:**
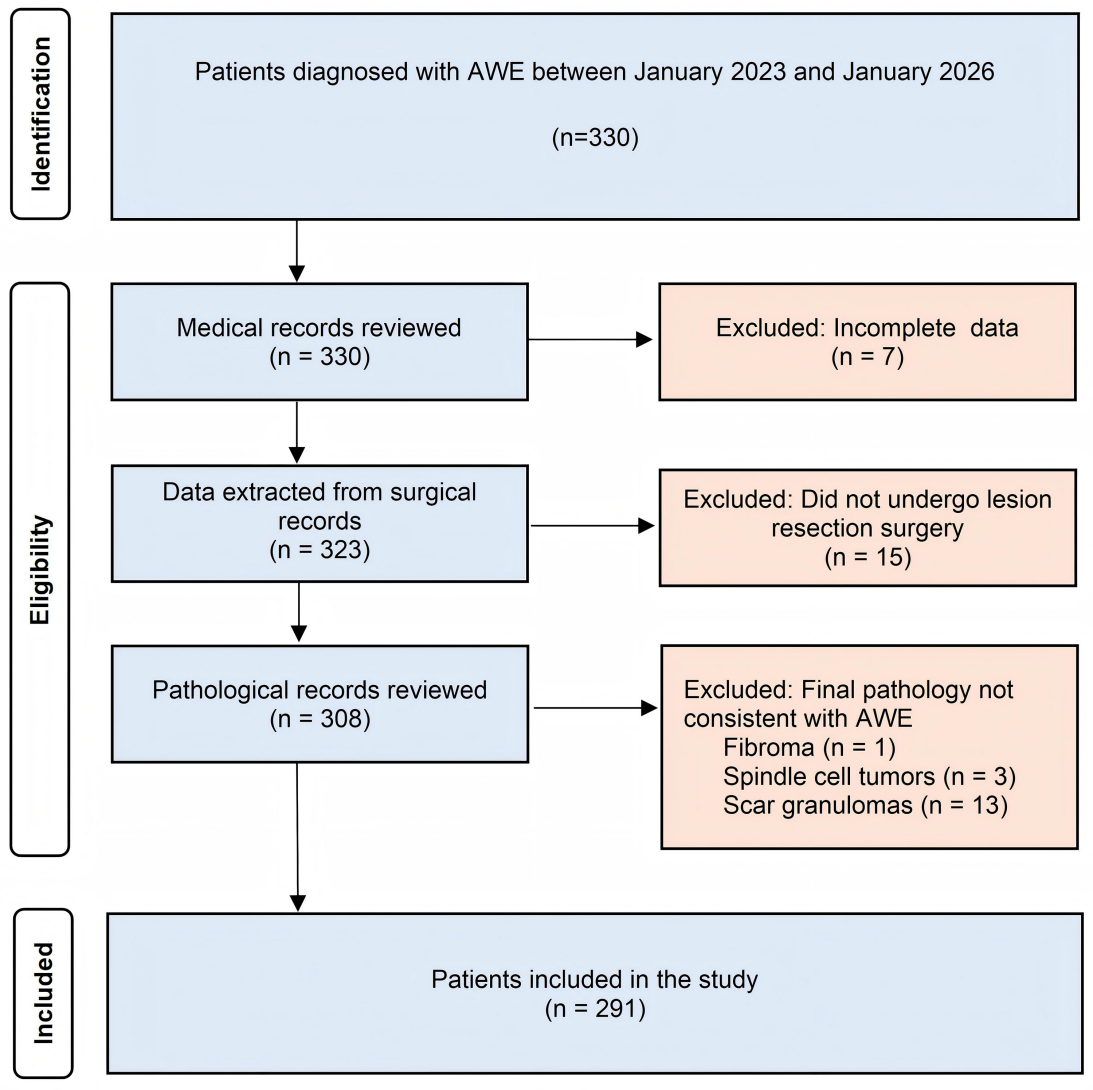
Flow chart of AWE patient enrollment and exclusion.

### 2.2 Data collection

The following data were collected from the electronic medical record system: age at diagnosis, body mass index (BMI), presenting symptoms, prior abdominal surgical history, latency period (defined below), clinical course, preoperative ultrasound reports, intraoperative findings, postoperative pathology, perioperative complications, and follow-up outcomes.

The maximum lesion diameter was measured on the surgical specimen immediately after excision, before specimen fixation, using a standard ruler. All surgeons followed the same measurement protocol. As this was a retrospective study based on routine clinical practice, ultrasound readers and surgeons were not blinded to each other's assessments; the operating surgeons were aware of the preoperative ultrasound findings.

### 2.3 Definitions and classification

Latency period: The interval from the last cesarean section, or from the last gynecologic surgery in patients without a history of cesarean section, to symptom onset. This definition was applied uniformly to all patients.

Index operation: The first AWE resection performed at our institution.

Postoperative recurrence: Reappearance of abdominal wall pain or a painful mass at the original surgical site at least 6 months after the index operation, with pathological confirmation of AWE upon reoperation.

Infiltration classification: The depth of infiltration was determined intraoperatively based on the deepest layer of involvement. According to previous studies (7, 9, 10), AWE was classified into three types: Type I (subcutaneous)—the lesion confined to the skin and subcutaneous fat, not reaching the anterior rectus sheath; Type II (fascial/muscular)—the lesion invading the rectus sheath and/or rectus abdominis muscle, without peritoneal involvement; Type III (peritoneal)—the lesion reaching the peritoneum or breaching the abdominal cavity.

For patients with multiple lesions, we selected the largest or deepest-infiltrating lesion as the representative for analysis.

### 2.4 Follow-up

Patients were followed up through outpatient visits or telephone interviews, with follow-up ending on July 15, 2026. Those without documented recurrence were censored at last follow-up. Patients who met the recurrence definition during follow-up were assigned to the recurrence group. The non-recurrence group comprised patients who underwent the index operation at our institution and had no recurrence during follow-up. Patients with a history of recurrence before cohort entry were excluded from both groups.

### 2.5 Statistical analysis

Statistical analyses were carried out using R (version 4.6.1). Normality of differences was tested by Shapiro–Wilk test. Continuous variables were expressed as mean ± SD and compared using t-test or ANOVA; non-normally distributed variables were expressed as median [IQR] and compared using Mann–Whitney U or Kruskal–Wallis H tests. Categorical variables were presented as frequencies (percentages) and compared using the chi-square test or Fisher's exact test (when expected frequencies were <5). Agreement between preoperative ultrasound and intraoperative measurements of maximum lesion diameter was assessed using the intra-class correlation coefficient (ICC; two-way random-effects, absolute agreement) and Bland–Altman analysis (13). This ICC model was selected because the same set of patients was assessed by two different measurement methods, and the results were intended to be generalizable to other raters. The performance of ultrasound for detecting myofascial invasion was evaluated against intraoperative findings. Sensitivity, specificity, predictive values, and accuracy were calculated with 95% CIs. Recurrence was analyzed using Kaplan–Meier method. *P* < 0.05 was considered significant.

## 3 Results

### 3.1 Baseline characteristics

A total of 330 AWE patients were identified during the study period, giving an estimated post-cesarean AWE incidence of 0.68%–0.80% at our institution, similar to recent database reports (14). After excluding those with incomplete clinical data or other reasons, 291 AWE patients were included. Based on the infiltration depth classification, there were 64 Type I cases (22.0%), 183 Type II cases (62.9%), and 44 Type III cases (15.1%). The most common presenting symptom was a palpable abdominal mass (238 patients, 81.8%), and symptoms related to the menstrual cycle were reported in 136 patients(46.7%). The median age at diagnosis was 34 (IQR 31–36), with a significant difference among the three groups (*P* = 0.030).

A history of CS was present in 290 patients (99.7%), of whom 25.8% (75/291) had undergone two or more cesarean deliveries; only one patient had no history of CS but had previously undergone laparoscopic myomectomy. The median time from last CS to diagnosis was 48 months (IQR 36–72), with a significant difference among the three types (*P* = 0.023); Type III patients had the longest median interval at 60 months (IQR 48–87). The median latency period and from symptom onset to diagnosis were 34 months (IQR 18–48) and 12 months (IQR 6–30), respectively, with no significant differences among the three types (*P* = 0.215 and *P* = 0.115, respectively).

To further explore the relationship between the number of CS and lesion characteristics, patients were stratified into the single CS group (*n* = 215) and the multiple CS group (*n* = 75). No statistically differences were observed between two groups with respect to infiltration depth distribution (*P* = 0.138), maximum lesion diameter (*P* = 0.101), or proportion of multiple lesions (*P* = 0.225). Figure 2 shows the cumulative distribution curves of three clinical time intervals, stratified by the number of prior CS (single vs. multiple). Two groups presented largely similar patterns across all three intervals, with no statistically significant differences (*P* = 0.110, *P* = 0.915, and *P* = 0.157, respectively).

**Figure 2:**
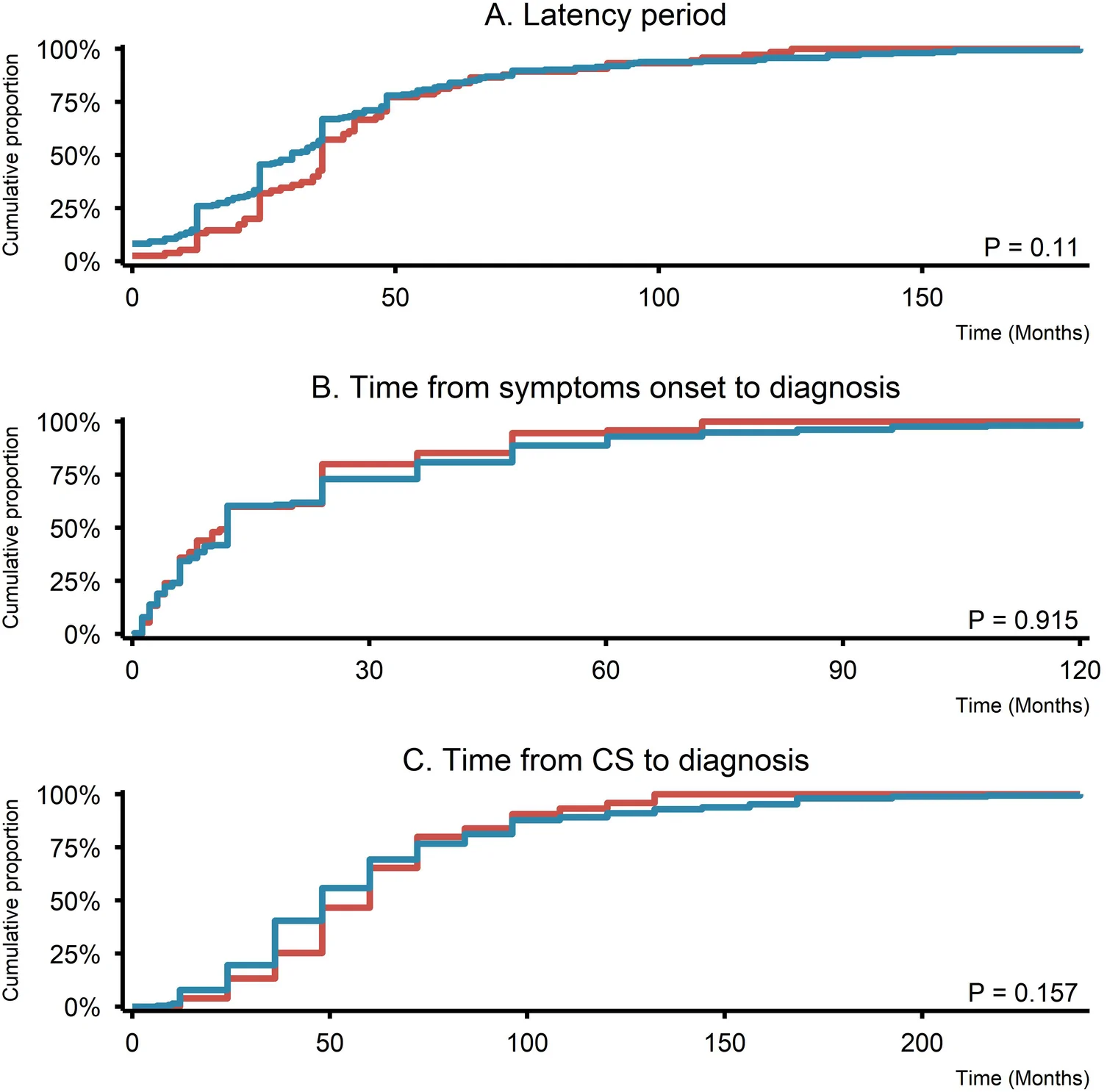
Cumulative distribution of the latency period **(A)**, from symptom onset to diagnosis **(B)**, and from CS to diagnosis **(C)**, stratified by the number of previous cesarean deliveries (1 vs. ≥ 2). The blue and red lines represent patients with one CS (*n* = 215) and two or more CSs (*n* = 75), respectively.

In our cohort, concomitant pelvic endometriosis was uncommon. Only 17 patients (5.8%) had ovarian endometriomas, and 2 patients (0.7%) had deep endometriosis.

### 3.2 Ultrasound findings and concordance with intraoperative findings

All patients underwent preoperative superficial ultrasound examination. Typical B-mode ultrasound findings included hypoechoic nodules with poorly defined borders, irregular contours(Figure 3A). Most lesions showed poor vascularity on Doppler imaging (Figure 3B). Among patients with positive ultrasound findings who underwent surgery, the positive predictive value of ultrasound for AWE was 94.5% (291/308). The 17 (5.5%) false-positive cases included scar granuloma (*n* = 13), spindle cell tumor (*n* = 3), and fibroma (*n* = 1).

**Figure 3:**
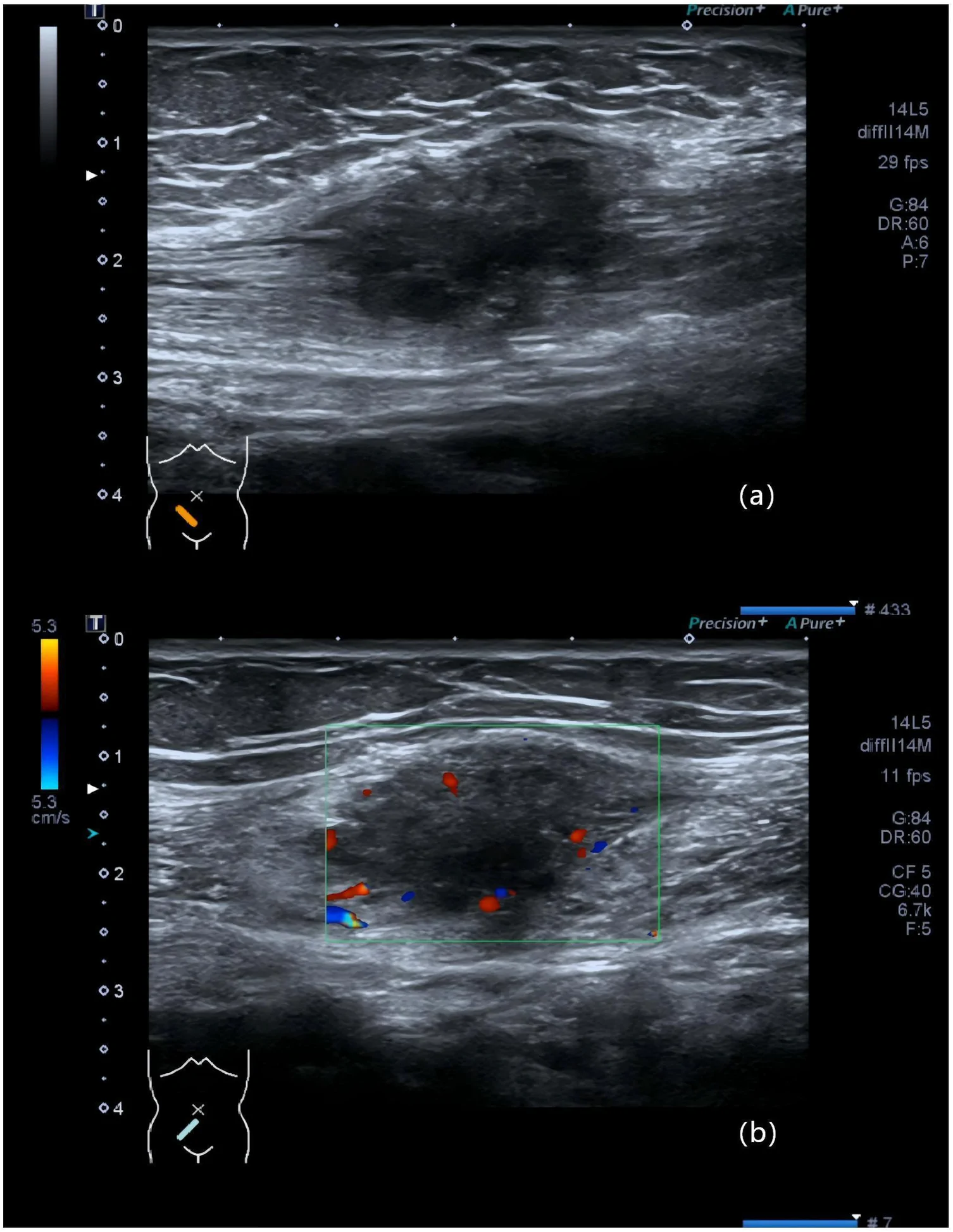
Grayscale and color Doppler ultrasound features of an abdominal wall endometriosis (AWE) lesion. **(a)** Grayscale ultrasound image showing a hypoechoic mass within the muscular layer, with ill-defined borders and an irregular shape. **(b)** Color Doppler image demonstrating sparse blood flow signals within and around the lesion. AWE, abdominal wall endometriosis.

In assessing the maximum diameter of the lesion, the ICC between preoperative ultrasound and intraoperative exploration measurements was 0.872 (95% CI: 0.788–0.916), indicating good overall agreement. In a further analysis stratified by depth of invasion, the ICCs for Types I, II, and III were 0.803, 0.883, and 0.794, respectively, suggesting that the agreement between ultrasound and surgical measurements was at an acceptable level across all types (Table 3). Differences were non-normally distributed (Shapiro–Wilk, *P* < 0.001). Therefore, a non-parametric Bland-Altman approach was used (13), with the median difference as the bias estimate and the 2.5th–97.5th percentiles shown at Table 3. Preoperative ultrasound slightly underestimated lesion diameter versus intraoperative measurement (median difference: −0.20 cm; 2.5th−97.5th percentile: −1.40 to 0.60 cm); only 5.8% (17/291) of data points fell outside these limits.

**Table 3:** Comparison of preoperative ultrasound and intraoperative measurements of maximum lesion diameter.

Group	Ultrasound, median [IQR], cm	Intraoperative, median [IQR], cm	Median difference, 2.5th–97.5th percentiles^a^^,^ cm	ICC (95% CI)
Overall (*n* = 291)	2.20 [1.80, 3.00]	2.50 [2.00, 3.00]	−0.20 [−1.40, 0.60]	0.872 (0.788, 0.916)
Type I (*n* = 64)	2.00 [1.40, 2.22]	2.00 [2.00, 3.00]	−0.20 [−1.19, 0.60]	0.803 (0.565, 0.899)
Type II (*n* = 183)	2.20 [1.72, 3.00]	2.50 [2.00, 3.00]	−0.11 [−1.17, 0.60]	0.883 (0.815, 0.922)
Type III (*n* = 44)	2.95 [2.48, 3.95]	3.00 [3.00, 5.00]	−0.20 [−2.17, 0.80]	0.794 (0.594, 0.893)

a For non-normally distributed differences, non-parametric Bland–Altman analysis was used (13).

Preoperative ultrasound was also used to assess whether lesions involved myofascial invasion. Using intraoperative findings as the gold standard, the positive predictive value for myofascial invasion in this surgical cohort was 88.8%, indicating that when ultrasound suggested myofascial involvement, the finding was generally reliable. However, the negative predictive value in this surgical cohort was notably low at 44.7%, corresponding to a false-negative rate of 22.9%, indicating that ultrasound frequently failed to detect invasion that was subsequently confirmed during surgery in our surgical cohort (Table 4). Agreement between ultrasound and intraoperative findings was poor (Cohen's kappa = 0.366, 95% CI: 0.241–0.490). The McNemar test revealed a statistical difference between the two methods (*P* < 0.001). Because only surgically treated patients were included, these estimates should be interpreted in light of verification and selection bias.

**Table 4:** Diagnostic performance of preoperative ultrasound for detecting myofascial invasion in AWE.

Preoperative Ultrasound	Intraoperative Finding (Myofascial Invasion)		Total
	Positive	Negative	
Positive	175 (TP)	22 (FP)	197
Negative	52 (FN)	42 (TN)	94
Total	227	64	291

Sensitivity = TP/(TP + FN) = 175/(175 + 52) = 77.1%; Specificity = TN/(TN + FP) = 42/(42 + 22) = 65.6%; Positive predictive value = TP/(TP + FP) = 175/(175 + 22) = 88.8%; Negative predictive value = TN/(TN + FN) = 42/(42 + 52) = 44.7%; Accuracy = (TP + TN)/Total = (175 + 42)/291 = 74.6%; False-negative rate = FN/(TP + FN) = 52/(175 + 52) = 22.9%. These estimates should be interpreted in light of the verification and selection bias inherent to this surgically confirmed cohort.

TP, true positive; FP, false positive; FN, false negative; TN, true negative.

### 3.3 Surgical findings and postoperative recurrence

With increasing depth of infiltration, intraoperative maximum lesion diameter showed a significant upward trend. The proportion of lesions with a maximum diameter ≥3 cm increased from 26.6% (17/64) in Type I to 44.8% (82/183) in Type II, and further to 84.1% (37/44) in Type III (*P* < 0.001). Solitary lesions accounted for 88.0% (*n* = 256), while multiple lesions accounted for 12.0% (*n* = 35), with no significant difference in distribution among the three types (*P* = 0.395).

The overall incidence of intraoperative mesh placement was 2.4% (*n* = 7), with no significant difference among three groups (*P* = 0.068). The overall incidence of postoperative complications (fever, poor healing, hernia, etc.) was 3.8% (*n* = 11), with Type III complications (13.6%) significantly higher than those in Type I (0%) and Type II (2.7%, *P* = 0.002). 94 patients (32.3%) received postoperative GnRH-a therapy. No significant difference was found in its use among the three infiltration types (*P* = 0.503).

Among the 291 included patients, 6 were referred after recurrence following initial surgery at other hospitals and were excluded from the recurrence analysis. The remaining 285 patients underwent the index operation at our institution. Of these, 13 were lost to follow-up (no follow-up data available after discharge) and were also excluded from the recurrence analysis; 272 with available follow-up data were included in the recurrence analysis. Of the 272 patients, 5 patients experienced recurrence and 267 were censored at the last follow-up. The recurrence rate was 1.8% (5/272). The median follow-up duration was 20.5 months (IQR 12–31.25) for the entire recurrence-analysis cohort (*n* = 272), 20 months (IQR 12–31) for the non-recurrence group, and 24 months (IQR 21–38) for the recurrence group.

Descriptive characteristics between the recurrence group (*n* = 5) and the non-recurrence group (*n* = 267) are shown in Table 5. BMI and maximum lesion diameter appeared numerically larger in the recurrence group, and the proportion of multiple lesions appeared higher. Kaplan–Meier curves for BMI, number of CS, maximum lesion diameter, lesion number, and infiltration type, together with the numbers at risk, censored, and events, are presented in Supplementary Materials 1. Kaplan–Meier analysis suggested a higher recurrence rate in patients with BMI ≥25 kg/m^2^. Given the very small number of recurrence events, all the above analyses should be regarded as highly exploratory and interpreted with caution, and no formal statistical comparison or Cox analysis was performed.

**Table 5:** Descriptive characteristics between the recurrence and non-recurrence groups.

Characteristic	Recurrence group (*n* = 5)	Non-recurrence group(*n* = 267)
BMI, kg/m^2^, median [IQR]	25.96 [21.26, 26.84]	21.30 [20.30, 23.52]
BMI ≥ 25 kg/m^2^, *n* (%)	3 (60.0%)	37 (13.9%)
≥2 CS, *n* (%)	2 (40.0%)	69 (25.8%)
Maximum diameter, cm, median [IQR]	4.1 [3.0, 4.9]	2.5 [2.0, 3.0]
Maximum diameter ≥ 3 cm, *n* (%)	4 (80.0%)	122 (45.7%)
Depth of infiltration, *n* (%)		
Type I (subcutaneous)	1 (20.0%)	57 (21.3%)
Type II (fascial/muscular)	2 (40.0%)	171 (64.1%)
Type III (peritoneal)	2 (40.0%)	39 (14.6%)
Multiple Lesion, *n* (%)	3 (60.0%)	28 (10.5%)

Given the very small number of recurrence events (*n* = 5), data are presented descriptively only; no formal statistical comparison was performed.

.

## 4 Discussion

Based on a cohort of 291 AWE cases, this study systematically analyzed clinical characteristics, the value of preoperative ultrasound, and postoperative recurrence patterns. Our key findings include: (1) Different infiltration depths are associated with distinct clinical features and surgical outcomes. Deeper infiltration was associated with older age at diagnosis, longer interval from CS to diagnosis, larger lesion size, higher rate of postoperative complications. (2) AWE was almost universally associated with a history of CS (99.7%), but the number of CS was not associated with lesion invasion depth, size, or multifocality; (3) Preoperative ultrasound showed good agreement with intraoperative measurements of lesion size (ICC=0.872), but had limited ability to detect myofascial invasion (positive predictive value 88.8%, false-negative rate 22.9%, negative predictive value 44.7%), suggesting that ultrasound carries a high risk of missing myofascial invasion and should be interpreted with caution. (4) The overall postoperative recurrence rate was low, and exploratory Kaplan–Meier analysis suggested a higher recurrence rate in patients with BMI ≥25 kg/m^2^. However, this finding should be regarded as hypothesis-generating given the limited number of events. Larger studies are needed to validate these results and to identify patients who may benefit from closer follow-up.

### 4.1 Clinical features and significance of classification

The classic triad of AWE—cyclic abdominal pain, a palpable mass, and a history of abdominal surgery (15)—remains a useful diagnostic guide. In our cohort, 46.7% of patients reported cyclic symptoms. The underlying mechanism involves ectopic endometrial tissue in the abdominal wall undergoing cyclic bleeding in response to hormonal fluctuations, leading to palpable mass or pain (2). Notably, we found that deep lesions (Type III) were more likely to present with abdominal wall pain than a palpable mass. This may be attributable to the more direct stimulation of sensory nerve endings in the deep fascia, muscle, or peritoneum by deep lesions, thereby making pain a more prominent presenting symptom. For patients with pain at a previous incision site but no palpable nodule, ultrasound or MRI should be considered to avoid misdiagnosis.

AWE is more commonly seen in women with a history of CS (2). In our cohort, 99.7% of patients had a prior cesarean delivery, and 25.8% had multiple CS. However, we found no association between the number of CS and lesion depth, size, multiplicity, or recurrence risk, consistent with previous reports (16). This suggests that there may not be a clear dose-response relationship between CS frequency and the occurrence or development of AWE; individual differences, surgical technique details, or other factors may play a more significant role (17). No prospective trials have evaluated AWE prevention. Intraoperative preventive measures—including abundant irrigation of incision sites, changing gloves before closure, and using a protective bag (during laparoscopy)—may help reduce the risk of iatrogenic endometrial implantation (2). To our knowledge, no study has specifically evaluated pharmacological prevention of AWE occurrence.

While retrograde menstruation is the leading theory for pelvic endometriosis, it does not adequately explain AWE, as most AWE lesions are extra-peritoneal (18). In our cohort, concomitant pelvic endometriosis was rare (ovarian endometriomas in 5.8%, DE in 0.7%), and its presence did not affect clinical course, consistent with the recent findings reported by Salmeri et al (4). Notably, in our study, the diagnosis of concomitant pelvic endometriosis depended on preoperative imaging and intraoperative findings. Because some patients presented predominantly with abdominal mass or pain, they underwent only superficial ultrasonography rather than further endometriosis-specific assessment. Moreover, laparoscopic surgery was not performed in all cases, and most patients with superficial lesions underwent local excision alone. Accordingly, a proportion of AWE cases with concomitant pelvic endometriosis may have remained undiagnosed. These considerations should be borne in mind when interpreting the relatively low coexistence rate in our cohort. Nonetheless, in light of both the low coexistence rate observed herein and the finding that concomitant pelvic endometriosis did not influence the clinical course, we propose that iatrogenic implantation alone is sufficient to initiate scar endometriosis. In contrast, spontaneous AWE, which is much rarer, is thought to arise from hematogenous or lymphatic spread of pelvic endometriosis (15).

One of the key findings of this study concerns the clinical significance of classification by depth of invasion. We classified lesions based on their depth of invasion into the abdominal wall as subcutaneous (Type I), fascial/muscular (Type II), and peritoneal (Type III). Type III lesions were associated with older age at diagnosis (*P* = 0.030), longer interval from cesarean section to diagnosis (*P* = 0.023), larger lesions (*P* < 0.001), and a significantly higher rate of postoperative complications (*P* = 0.002), consistent with the findings in recent researches (8, 10). This suggests that the depth of infiltration is not only a marker of disease severity but is also directly related to surgical difficulty and perioperative management strategies. And accurate preoperative classification is of great value in guiding surgical planning.

### 4.2 Value and limitations of preoperative ultrasound

Superficial ultrasound, owing to its non-invasive nature, convenience, and high resolution, is the first-line imaging modality for abdominal wall masses (19). If an abdominal wall mass with indistinct borders (or jagged edges), a solid nodule with hypoechoic or mixed echogenicity, internal hyperechoic foci or streaks, and a surrounding hyperechoic halo is detected, AWE should be included in the differential diagnosis; Suspicion should be heightened if the lesion is located near a cesarean section scar (5, 20). In this study, ultrasound achieved a positive predictive value of 94.5% in patients with suspected AWE. Because only patients who underwent surgery and had pathologically confirmed AWE were included, the sensitivity, specificity, and negative predictive value of ultrasound could not be reliably estimated due to verification/selection bias. Preoperative ultrasound demonstrated high agreement with the maximum diameter of the lesion as measured surgically (ICC = 0.872). But it is worth noting that both our study and another study (7) found that the maximum diameter of the lesion as assessed by preoperative ultrasound was smaller than that measured intraoperatively, and this difference was statistically significant. This may be related to the difficulty of clearly visualizing the infiltration margins using two-dimensional ultrasound.

Accurate preoperative assessment of the depth of lesion infiltration (involving the fascia or rectus muscle or not) into the abdominal wall is equally important (21). Superficial subcutaneous lesions are generally straightforward to excise, whereas lesions involving the rectus sheath or muscle may require wider resection and sometimes mesh implantation, increasing surgical risk and recovery time (7, 10). Furthermore, deeper lesions may require different surgical approaches. Studies have found that for Type II or Type III lesions, a laparoscopic approach facilitates early postoperative recovery (22) and allows for concurrent management of pelvic diseases (23). However, ultrasound has limitations in detecting subtle myofascial invasion. In our cohort, the positive predictive value for detecting myofascial invasion was 88.8%, whereas the negative predictive value was only 44.7%, with a false-negative rate of 22.9%. This indicates that a negative ultrasound finding could not reliably exclude myofascial invasion. Analysis revealed that in most false-negative cases, the lesions were primarily located in the subcutaneous fat layer; however, intraoperative exploration found involvement of the anterior sheath of the rectus abdominis muscle, and ultrasound failed to identify this subtle fascial involvement. This may be related to the limited resolution of two-dimensional ultrasound in visualizing fascial layers. These estimates should be interpreted in light of the verification and selection bias inherent to this surgically confirmed cohort. The integration of new ultrasound technologies may aid in preoperative lesion localization. A study by Wozniak et al. found that elastography substantially enhanced the accuracy of ultrasound assessment of AWE invasion depth (24). When necessary, MRI can be used as a supplementary modality to guide the selection of the surgical approach and the preparation of patches.

### 4.3 Recurrence and malignant transformation

The postoperative recurrence rate in this cohort was 1.8%, lower than the 5.3% reported in a recent systematic review (4), possibly due to the shorter follow-up duration in our study. BMI and maximum lesion diameter appeared numerically larger in the recurrence group, and the proportion of multiple lesions appeared higher. Kaplan–Meier analysis suggested a higher recurrence rate in patients with BMI ≥25 kg/m^2^. Given the extremely small number of events, these findings are hypothesis-generating and require confirmation in larger studies. Biologically, adipose tissue-derived estrogen and leptin are elevated in obesity and can promote the proliferation and invasiveness of ectopic endometrial cells (25), which may partly explain a potential association with recurrence. Larger lesions may be more difficult to excise completely due to extensive fibrosis and indistinct margins, which may result in residual microscopic foci. We hypothesize that the multifocal nature of known lesions may reflect multifocal implantation of endometrial tissue within the abdominal wall incision: some microscopic deposits may not be visible at the time of initial surgery and may gradually grow over months or years to become clinically apparent. Therefore, patients with multiple lesions may have a higher risk of recurrence due to residual microscopic foci that were not detectable during the index operation. However, these hypotheses require further investigation. Previous studies have suggested that complete resection of the lesion and surrounding fibrotic tissue may reduce the risk of recurrence (7, 26). Currently, open surgery combined with wide local excision with a 1 cm margin is considered the optimal treatment for AWE (2).

Medical therapy typically alleviates symptoms but does not eradicate the disease. Previous studies on the effect of postoperative hormonal therapy on AWE recurrence have yielded inconsistent results. Several studies published in the past five years have suggested that postoperative hormonal therapy is not significantly associated with recurrence (7, 27, 28). Conversely, an earlier study suggested that combining surgery with GnRH-a or Danazol might decrease recurrence (29). However, these studies were limited by small sample sizes and retrospective designs. Prospective randomized trials are needed to evaluate the role of hormonal therapy in preventing recurrence after AWE resection.

Furthermore, although malignant transformation of AWE is extremely rare, one patient in our cohort developed malignancy 3 years after AWE resection. This 46-year-old woman had only one prior cesarean section 25 years earlier. She underwent excision of an abdominal mass at an outside hospital 5 years ago, with pathology confirming AWE. Two years ago, she presented with a rapidly growing, persistently painful mass at the same site; re-excision confirmed clear cell carcinoma. She then received regular platinum-based chemotherapy at our institution, with no recurrence during follow-up. Clear cell carcinoma is the most common histological subtype of malignant transformation arising from AWE, yet only approximately 30 cases have been reported (2). Malignant transformation of AWE typically presents as a mass around a previous abdominal scar, often accompanied by abdominal pain (30). Abdominopelvic CT and MRI are commonly used to define the extent of disease involvement. For patients approaching menopause or receiving estrogen replacement therapy, vigilance for malignant transformation is warranted when rapid growth, atypical imaging, or markedly elevated CA-125 is observed (7, 31).

### 4.4 Limitations

Our study has several limitations. First, given the retrospective design, information bias cannot be ruled out. Second, because AWE is relatively rare, follow-up durations were heterogeneous, which may have underestimated the true recurrence rate. Third, surgeries were performed by multiple gynecologists, and variations in surgical technique may have influenced outcomes. Fourth, central blinded review of ultrasound and surgical findings was not feasible, which may have introduced observer bias. Fifth, Surgical margin status was not systematically recorded. Therefore, we could not evaluate the association between margin status and recurrence. And centralized pathology review was not performed. Additionally, the number of recurrence events was extremely small, thus the recurrence analysis should be regarded as hypothesis-generating and requires validation in larger studies. Finally, as a single-center study from a tertiary referral hospital, our cohort may not be representative of the general AWE population, and our findings may not be directly generalizable to other settings.

## 5 Conclusions

Abdominal wall endometriosis occurs more frequently among women with a history of cesarean section, though the number of cesarean deliveries does not correlate with lesion depth, size, or multiplicity. Deeper infiltration is linked to increased surgical complexity and postoperative complications. Ultrasound is reliable for measuring lesion size but limited in detecting myofascial invasion. The observed recurrence rate was low after surgical excision in this cohort. Larger prospective studies are needed to identify patients at higher risk who may need closer follow-up.

## Data Availability

The original contributions presented in the study are included in the article/Supplementary Material, further inquiries can be directed to the corresponding author.
