# Supplementary material for: Post-surgical abdominal wall endometriosis: a retrospective cohort study on clinical features, ultrasound accuracy and recurrence
Source: Front Med (Lausanne). 2026 Oct 29;13:1969114. doi: 10.3389/fmed.2026.1969114 (PMC13754834; doi:10.3389/fmed.2026.1969114)
Supplement: Supplementary file 1 [file Datasheet1.pdf]

Supplementary Materials 1. *Kaplan–Meier curves with numbers at risk, censored, and events for recurrence after surgical excision of AWE.*

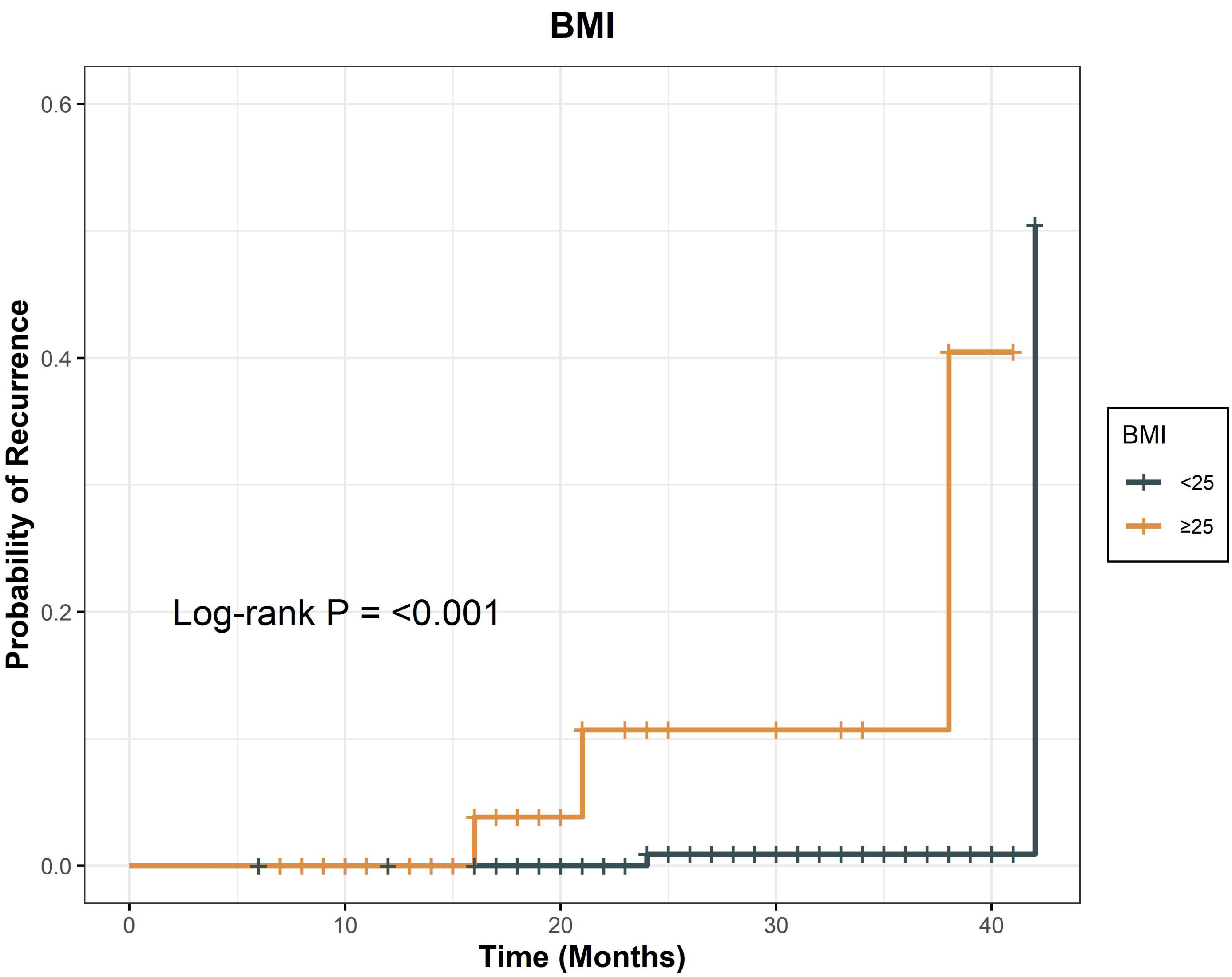

Time (mo)	**0**	**10**	**20**	**30**	**40**	**42**
**< 25**						
At risk	232	191	122	72	10	0
Censored	0	41	110	159	221	230
Events	0	0	0	1	1	2
**≥ 25**						
At risk	40	31	14	6	1	0
Censored	0	9	25	32	36	37
Events	0	0	1	2	3	3

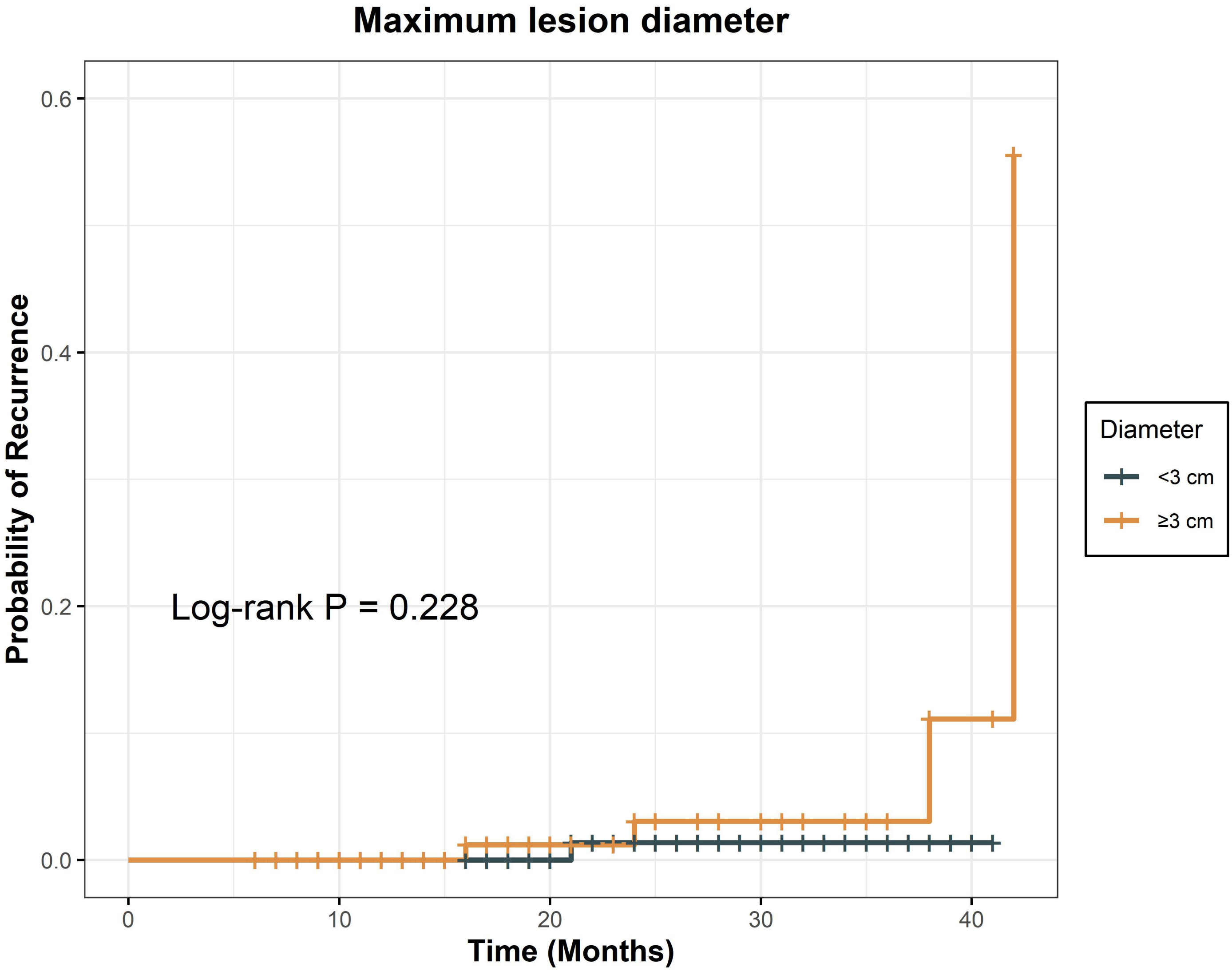

Time (mo)	**0**	**10**	**20**	**30**	**40**	**42**
**< 3cm**						
At risk	146	117	73	49	5	0
Censored	0	29	73	96	140	145
Events	0	0	0	1	1	1
**≥ 3cm**						
At risk	126	105	63	29	6	0
Censored	0	21	62	95	117	122
Events	0	0	1	2	3	4

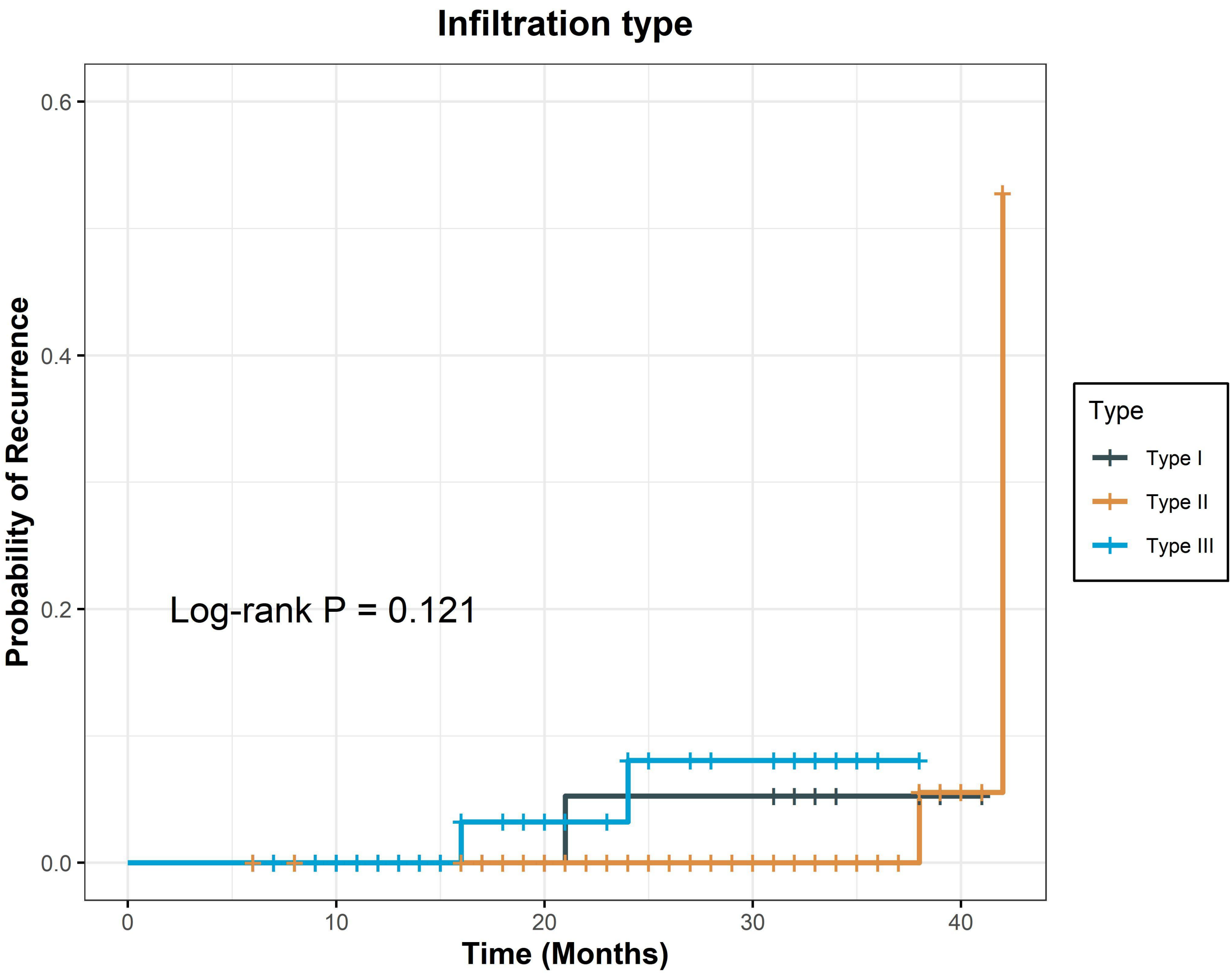

Time (mo)	**0**	**10**	**20**	**30**	**40**	**42**
Type I						
At risk	58	33	19	18	2	0
Censored	0	25	39	39	55	57
Events	0	0	0	1	1	1
Type II						
At risk	173	153	93	48	9	0
Censored	0	20	80	125	163	171
Events	0	0	0	0	1	2
Type III						
At risk	41	36	24	12	0	0
Censored	0	5	16	27	39	39
Events	0	0	1	2	2	2

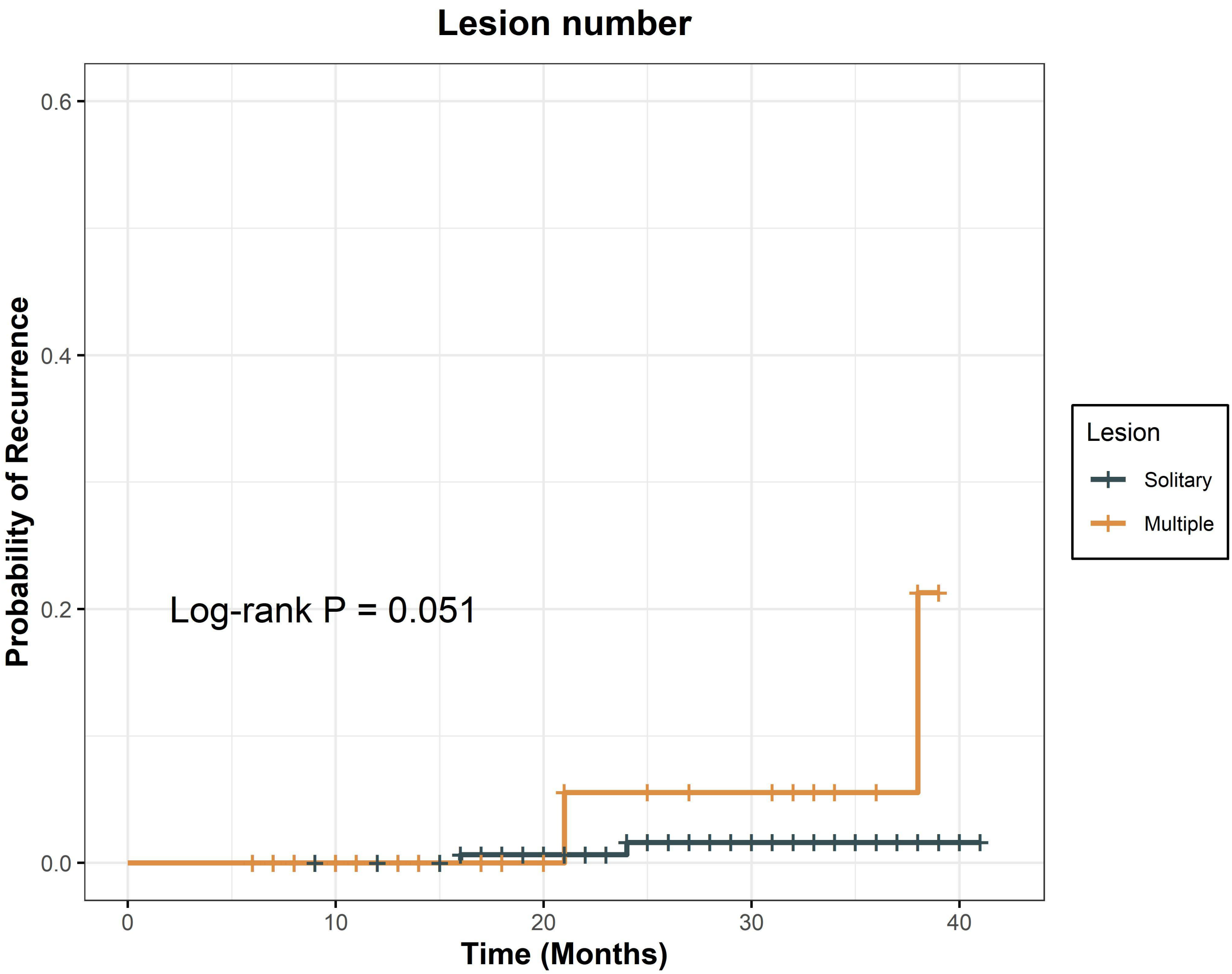

Time (mo)	**0**	**10**	**20**	**30**	**40**	**42**
Solitary						
At risk	241	196	118	64	9	0
Censored	0	45	122	175	230	239
Events	0	0	1	2	2	2
Multiple						
At risk	31	26	18	14	2	0
Censored	0	5	13	16	27	28
Events	0	0	0	0	2	3

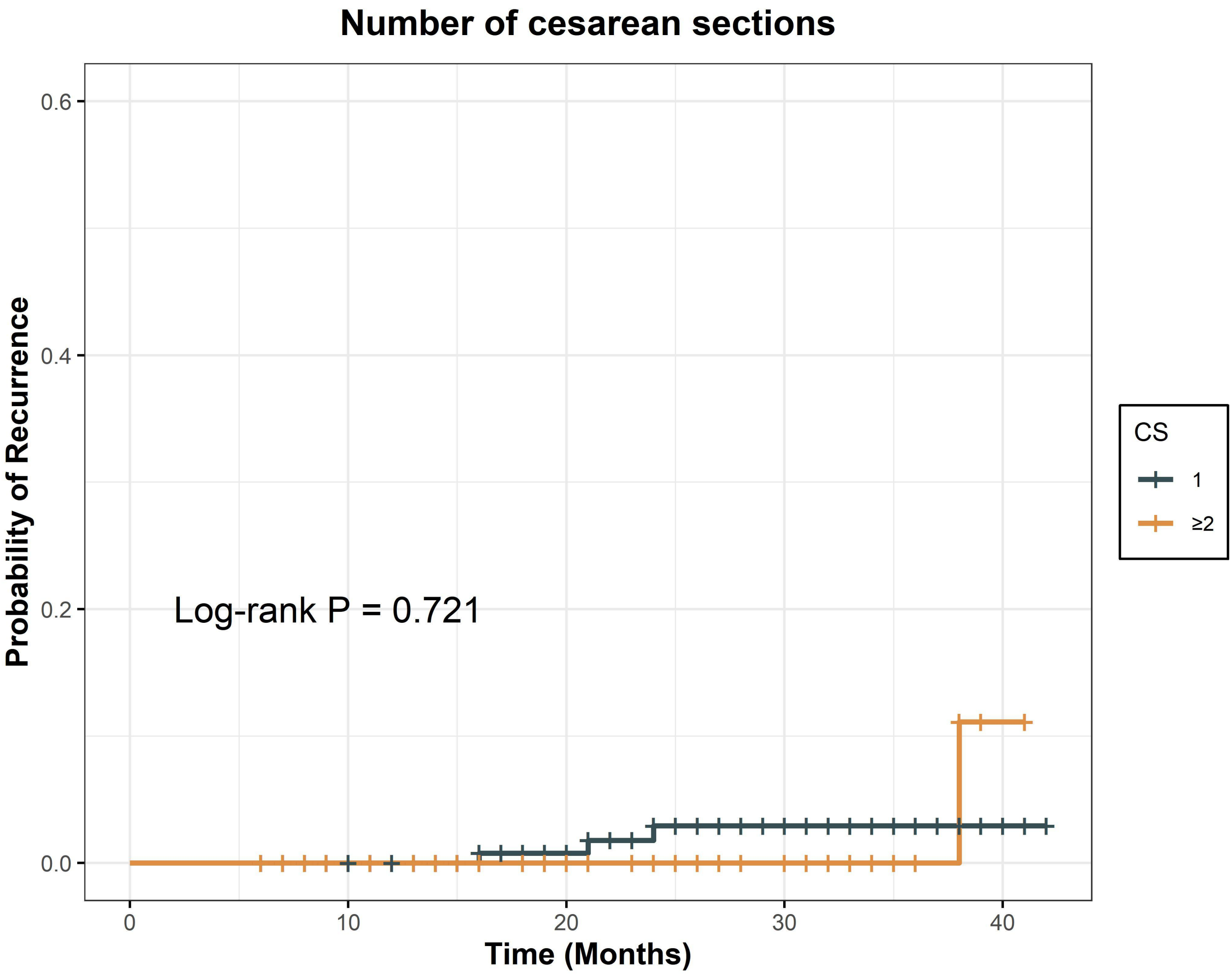

Time (mo)	**0**	**10**	**20**	**30**	**40**	**42**
1 CS						
At risk	201	161	99	57	9	0
Censored	0	40	101	141	189	198
Events	0	0	1	3	3	3
≥ 2 CS						
At risk	71	61	37	21	2	0
Censored	0	10	34	50	68	69
Events	0	0	0	0	1	2
